# A metagenomic snapshot of metabolic potentials in wintertime eutrophic sediments of Lake Taihu inferred from metagenome-assembled genomes

**DOI:** 10.3389/fmicb.2026.1929411

**Published:** 2026-09-28

**Authors:** Qi Wei, Yajie Li, Aidong Ruan

**Affiliations:** 1 College of Hydrology and Water Resources, Hohai University, Nanjing, China; 2 PowerChina Guiyang Engineering Corporation Limited, Guiyang, China

**Keywords:** biogeochemical cycling, horizontal gene transfer, Lake Taihu sediments, metagenome-assembled genomes, microbial metabolic potential, secondary metabolite biosynthetic gene clusters

## Abstract

**Introduction:**

Understanding the metabolic potential of microbial communities in eutrophic lake sediments at the genome-resolved level is essential for predicting nutrient cycling and stress responses in contaminated freshwater ecosystems.

**Methods:**

In this study, we reconstructed 206 dereplicated metagenome-assembled genomes (MAGs) from sediment cores collected across four regions of Lake Taihu.

**Results:**

These dereplicated MAGs spanned 34 classified phyla and 72 classified genera, with 97.6% unclassified at the species level, indicating substantial genomic novelty. The cyanobacterial MAGs harbored diverse secondary metabolite biosynthetic gene clusters, suggesting a genomic basis for potential adaptive traits. Across all dereplicated MAGs, the highest biosynthetic potential for secondary metabolites was observed in Chloroflexota and Pseudomonadota. Putative antibiotic resistance genes were predominantly represented by glycopeptide resistance determinants (vanT/vanW) and resistance-nodulation-division (RND) efflux pumps, which together accounted for 86.1% of the detected resistome. Additionally, 490 predicted horizontal gene transfer (HGT) events were identified, with transferred genes being most prevalent in energy metabolism (12.4%), translation (10.8%), and transcriptional regulation (7.2%).

**Discussion:**

This genome-resolved survey provides a foundation for understanding the metabolic and genomic potential of sediment microbiomes in eutrophic freshwater systems.

## 1 Introduction

Cyanobacterial blooms in eutrophic lakes represent one of the most pressing global water quality challenges, with Lake Taihu serving as a prominent model system. In the Taihu Basin, decades of rapid industrialization and urbanization have led to chronic external nutrient loading, which has pushed the lake far beyond its eutrophication threshold (Wei et al., 2024). However, while traditional management measures such as pollution source control and ecological water diversion have achieved short-term reductions in surface water nutrient concentrations (Qin et al., 2019), the persistent recurrence of blooms indicates that merely focusing on the single “nutrient–algae” relationship is insufficient. The shallow depth (average < 2 m) of Lake Taihu exacerbates sediment resuspension and internal nutrient release, and summer conditions—including high temperatures (25–35 °C) and low wind speeds—promote the aggregation of buoyant cyanobacteria (Yan et al., 2024). These physical and chemical factors create a dynamic backdrop for microbial processes, yet the mechanisms by which microorganisms mediate ecosystem resilience and feedback remain poorly understood.

The biogeochemical cycling of carbon, nitrogen, sulfur, and other elements in lake sediments is largely mediated by microbial communities (Wu et al., 2018). However, the functional potential of sediment microbial communities has long remained elusive due to the limitations of culture-based methods. Recent advances in metagenomics, particularly the reconstruction of metagenome-assembled genomes (MAGs), have revolutionized our ability to explore “microbial dark matter”—the vast majority of uncultivated microorganisms (Ma et al., 2023). MAGs provide a powerful lens for deciphering metabolic networks and ecological interactions within complex communities (Cheng et al., 2024). For instance, studies have successfully used MAGs to investigate metabolic cross-feeding in planktonic communities (Giordano et al., 2024), to assess microbial roles in sulfate reduction (Hu et al., 2023), and to reveal the coupling of carbon–nitrogen–sulfur cycles in sedimentary environments (Chen et al., 2024). Notably, a MAG-based study in Lake Taihu reconstructed 161 high-quality genomes from water samples, shedding light on cyanobacterial defense systems and energy pathways (Zhu et al., 2021). Despite this progress, comprehensive genome-resolved analyses that integrate the diverse metabolic and adaptive strategies of sediment microbiomes, especially under eutrophic stress, remain scarce.

The success of microorganisms in dynamic sedimentary environments relies on multiple adaptive strategies that extend beyond core metabolism. Secondary metabolism equips microorganisms with a chemical arsenal, including antibiotics, siderophores, and pigments, which mediate survival strategies such as competition, biofilm formation, and stress tolerance (He et al., 2019). Although cultured cyanobacteria are known to produce structurally complex and highly bioactive natural products (Dittmann et al., 2015), the secondary metabolic potential of the uncultivated majority in sediments remains a largely unexplored frontier. Complementary to chemical defense is the clustered regularly interspaced short palindromic repeats (CRISPR)-CRISPR-associated (Cas) immune system, an adaptive immune system in bacteria and archaea that provides sequence-specific immunity against phages and plasmids (Koonin and Makarova, 2019). The interplay between viral pressure and horizontal gene flow is a key determinant of microbial community structure, yet the distribution and ecological significance of CRISPR–Cas systems in virus-rich sediments of eutrophic lakes remain poorly understood.

Concurrently, the resistome—the collection of antibiotic resistance genes (ARGs)—has become a pervasive environmental pollutant, with ARGs spreading through mobile genetic elements across aquatic ecosystems (Allison and Martiny, 2008). Eutrophication has been shown to promote horizontal transfer of ARGs in microalgal-bacterial symbiotic systems, leading to increased complexity in bacterial interaction networks (You et al., 2024), suggesting that nutrient enrichment and antibiotic pollution may co-select for resistant and competitive microbial lineages. Horizontal gene transfer (HGT), the vehicle for such gene exchange, is a key mechanism of bacterial evolution, facilitating rapid acquisition and dissemination of antibiotic resistance among microbial populations (Soucy et al., 2015). HGT is recognized as a key mechanism in cyanobacterial evolution and may be associated with the competitive success of cyanobacteria during bloom events. Finally, the pangenome—the complete set of genes within a species—encompasses the genetic diversity that fuels adaptability: core genes maintain essential functions, while accessory genes reflect niche-specific adaptations (Rouli et al., 2015). Pangenomic analyses of *Microcystis* have revealed substantial genetic polymorphisms linked to toxicity and ecotype differentiation (Humbert et al., 2013), yet the adaptive significance of this genetic architecture in sediment environments remains to be fully elucidated.

Although each of these research fronts has advanced independently, they remain largely disconnected. There is a critical lack of a unified, genome-resolved framework that integrates secondary metabolism, immune defense, resistance potential, gene flow, and population genomics to explain the ecological success of microbial communities—particularly cyanobacteria—in eutrophic lake sediments. Such an integrated perspective is essential for moving beyond descriptive metagenomics toward predictive understanding of ecosystem function and for identifying novel targets for bloom mitigation.

To address this gap, we performed metagenomic sequencing of sediment cores from four hydrologically distinct regions of Lake Taihu and successfully reconstructed 206 dereplicated MAGs. The central question of this study is: What are the metabolic potentials of microorganisms in the wintertime eutrophic sediments of Lake Taihu, as inferred from the recovered MAGs? To address this question, we pursued four integrated sub-objectives: (1) to characterize the phylogenetic novelty and spatial distribution of these MAGs; (2) to elucidate the modular specialization underlying carbon-nitrogen-sulfur biogeochemical cycling; (3) to explore environmental adaptation mechanisms, specifically focusing on secondary metabolite biosynthesis and CRISPR-Cas immune defenses; and (4) to assess the resistome landscape and HGT-associated evolutionary dynamics. Together, this genome-resolved framework advances our understanding of microbial life in eutrophic sediments and provides a molecular foundation for sustainable lake management and bioremediation strategies.

## 2 Materials and methods

### 2.1 Study area and sample collection

Four representative regions of Lake Taihu (Dapu, Xu Bay, Gong Bay, and Central Lake) were selected as sampling sites in December 2017, a winter period with little hydrological disturbance (Figure 1). Sediment cores were collected vertically *in situ* using a core sampler. The sampling coordinates were as follows: Dapu (119°57’19.63”E, 31°17’55.58”N), Xu Bay (120°24’20.17”E, 31°10’14.00”N), Gong Bay (120°20’45.54”E, 31°23’10.02”N), and the Central Lake (120°08’11.75”E, 31°08’34.22”N). Each sediment core was sectioned at 5 cm intervals. Cores collected from Gong Bay and Xu Bay, with a length of 50 cm, were divided into 10 segments each, while those from Dapu and the Central Lake, measuring 40 cm in length, yielded 8 segments each. This resulted in a total of 36 depth sections, enabling comprehensive analysis of both vertical stratification and spatial heterogeneity across the sediment profile.

**Figure 1:**
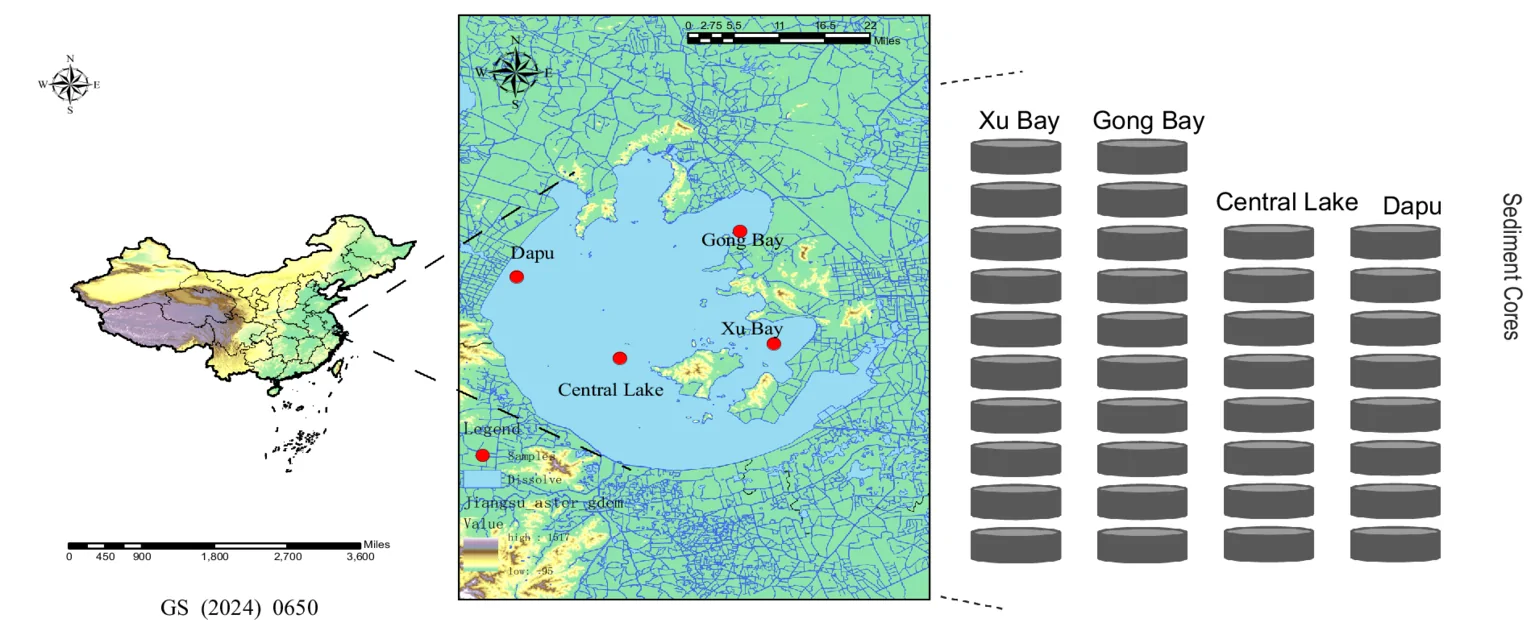
Sampling sites and methods in Lake Taihu.

For sample processing, different preservation strategies were employed depending on the intended analysis. Physicochemical analyses were performed on subsamples stored at −20 °C. For metagenomic analysis, subsamples underwent freeze-drying, homogenization, and sieving. Unprocessed subsamples were also archived at −80 °C to ensure material availability for future analyses.

The gravimetric water content (w, %) was determined by oven-drying at 105 °C to constant weight with a precision of ±0.1 mg. For grain-size analysis, a laser diffraction particle size analyzer (LS13320, Beckman Coulter, United States) was used. A 1 g sample was treated with H₂O₂ (30%, 60 °C) to remove organic matter and with 0.1 M HCl for decalcification, followed by particle dispersion using Na₆O₁₈P₆ as a dispersant. The particle size distribution spectrum (0.02–2000 μm) was obtained using the Fraunhofer model, with a coefficient of variation (CV) < 3% for reproducibility. Accuracy was validated against a national standard reference material (GBW07424). For chemical constituents, ammonium (NH₄^+^-N, mg/kg) and nitrate (NO₃^−^-N, mg/kg) were determined following the Chinese national environmental protection standard HJ 634–2012. These ions were desorbed from the sediment using 1 M KCl extraction (liquid-to-solid ratio 5:1, shaking for 30 min), thereby avoiding interference from organic nitrogen mineralization. Total phosphorus (TP, mg/kg) was extracted using HNO₃–HF-HClO₄ digestion, followed by colorimetric determination with the molybdenum blue method by measuring absorbance at 700 nm. Total organic carbon (TOC, g/kg) was determined by oxidizing organic carbon with K₂Cr₂O₇-H₂SO₄, and absorbance was measured at 585 nm to calculate TOC content. All measured data are provided in the Supplementary Table S1.

### 2.2 Metagenomic sequencing, assembly, and quality control of sediment microbial DNA

DNA was extracted from sediment subsamples using the TGuide S96 Magnetic Bead-based Soil/Fecal Genomic DNA Extraction Kit following the manufacturer’s protocol. DNA purity and integrity were assessed using a NanoDrop 2000 spectrophotometer and a Qubit 3 fluorometer and verified by 1.5% agarose gel electrophoresis (Tanon EPS600). Sequencing libraries were constructed using the VAHTS Universal Plus DNA Library Prep Kit on the Illumina NovaSeq 6,000 platform, with fragment size selection (350 ± 50 bp) and paired-end sequencing (PE150). Raw reads were processed with fastp v0.23.1 (Chen et al., 2018) for quality trimming (−5 -W 50 -M 20 -l 60 -n 0 -g -A). Quality control statistics before and after filtering are provided in the Supplementary Table S1. To remove potential human-derived contamination, reads were aligned to the human reference genome (GRCh38) using bowtie2 v2.2.4 (Langmead and Salzberg, 2012) (parameters: --seed 123,456 -I 200 -X 1000 --un-conc-gz), yielding 1.25 TB of clean data. Each of the 36 sediment subsamples was assembled independently using MEGAHIT v1.2.9 (Li et al., 2016) with default k-mer parameters (k-min = 27, k-max = 127). Contigs shorter than 300 bp were discarded to minimize interference from degraded DNA, and assembly quality was assessed with QUAST v5.2.0 (Gurevich et al., 2013). For each subsample, assembly quality metrics are provided in the Supplementary Table S1.

### 2.3 Metagenome-assembled genomes reconstruction and analysis

In this study, genome binning of sediment microbiomes was performed using MetaWRAP v1.3.2 (Uritskiy et al., 2018), which integrated MaxBin2 (Wu et al., 2016), CONCOCT (Alneberg et al., 2014), and MetaBAT2 (Kang et al., 2019). This process yielded a total of 460 raw bins from all subsamples. These raw bins were subsequently refined and filtered using CheckM, retaining only those with >50% completeness and <10% contamination (Parks, 2014). Redundancy was removed using dRep v3.4.5 (Olm et al., 2017) at a 95% average nucleotide identity (ANI) threshold, yielding 206 dereplicated MAGs. Taxonomic annotation was subsequently assigned using GTDB-Tk v2.3.2 (Chaumeil et al., 2022). MAG quality statistics are provided in the Supplementary Table S1. A phylogenetic tree of the 206 dereplicated MAGs was constructed using PhyloPhlAn v3.0.60 (Asnicar et al., 2020) in diversity and accurate modes. Clean reads were mapped to the metagenome-assembled genomes using CoverM v0.6.1 (https://github.com/wwood/CoverM) with thresholds of 95% nucleotide identity and 75% alignment coverage, and abundance was calculated as reads per kilobase per million mapped reads (RPKM). The phylogenetic tree was visualized using the interactive Tree of Life (iTOL) online tool (Letunic and Bork, 2024). Protein-coding genes were predicted from the metagenome-assembled genomes using Prodigal v2.6.3 (Hyatt et al., 2010). Functional annotation was performed using eggNOG-mapper v2.1.6 (Cantalapiedra et al., 2021) against the eggNOG v5.0.2 database (Huerta-Cepas et al., 2019) with DIAMOND (E-value ≤1e-3) and HMMER3 searches. To characterize the metabolic potential and biogeochemical functional profiles of the microbial community, we used METABOLIC v4.0 (Zhou et al., 2022). To quantify the relative importance of specific metabolic functions within the community, METABOLIC calculates a metabolic weight (MW) score for each function. The MW score is a composite metric that reflects the functional weight of a given metabolic function within the whole microbial community, integrating both the prevalence of the function across community members and the abundance (coverage) of the genomes harboring that function. MW scores were calculated for each metabolic pathway as the sum of normalized abundances of all genes belonging to that pathway, divided by the total community abundance. The percentage contribution of each phylum to a given pathway was calculated as the sum of MW scores for all dereplicated MAGs assigned to that phylum, divided by the total MW score for that pathway across all dereplicated MAGs, multiplied by 100. In addition, we used DRAM v1.3.5 (Shaffer et al., 2020) with default parameters against the UniRef90, PFAM, and dbCAN databases to annotate the metabolic functions of the dereplicated MAGs. Subcellular localization of proteins was predicted using DeepLocPro v1.0 to distinguish cytoplasmic, periplasmic, and extracellular proteins.

Secondary metabolite biosynthetic gene clusters (BGCs) were identified using AntiSMASH v7.1 (Blin et al., 2023) against the MIBiG v2.0 database (Kautsar et al., 2020). Gene cluster families (GCFs) were subsequently constructed with BiG-SCAPE v1.1.0 (Navarro-Muñoz et al., 2020) using a 0.3 sequence similarity threshold. CRISPR-Cas systems were detected using CRISPR-CasTyper (Russel et al., 2020). HGT events were identified with MetaCHIP v1.10.13 (Song et al., 2019) through phylogenetic conflict analysis. All other parameters were kept at their default values as specified in the MetaCHIP documentation.^1^ Briefly, the best-match approach used BLASTN with default E-value and sequence identity thresholds; phylogenetic reconciliation was performed using the embedded workflow to infer gene trees and species trees, with donor-recipient directionality determined by the phylogenetic placement of the transferred gene relative to the species tree. “Recent transfer” was defined following the MetaCHIP default criteria, which classify events based on genetic divergence levels. ARGs were predicted using RGI v6.0.3 against the CARD v3.2.9 database (Alcock et al., 2022) with hits retained at a Best_Hit_Bitscore ≥ 50, which was the strict cutoff recommended by RGI. The analysis was performed with DIAMOND for efficient alignment using the following parameters: --input_type protein, −-clean, and --alignment_tool DIAMOND.

### 2.4 Statistical analysis

All statistical analyses were performed using R (version 4.3.3). Alpha diversity indices (Shannon diversity and Chao1 richness) were calculated using the estimateR function from the R package vegan. Beta diversity was assessed based on Bray–Curtis dissimilarity using the vegdist function in vegan and visualized via principal coordinates analysis (PCoA) using the cmdscale function from the R package stats. Statistical significance of spatial variations (among sampling regions and sediment depths) was determined using permutational multivariate analysis of variance (PERMANOVA) via the adonis2 function in vegan, with 999 permutations. Abundance plots for MAGs and functional genes involved in carbon, nitrogen, and sulfur cycling were generated using the R package ggplot2 (v3.4.4). Final figure layouts were refined in Adobe Illustrator 2021 for labeling and panel assembly.

## 3 Results

### 3.1 Diversity and distribution of sediment metagenome-assembled genomes

In this study, metagenomic data from 36 depth sections of Lake Taihu were used. After redundancy removal with dRep and quality filtering (completeness ≥ 50%, contamination < 10%), 206 dereplicated MAGs were successfully reconstructed, including 135 bacterial MAGs (65.5%) and 71 archaeal MAGs (34.5%) (Figure 2). Phylogenetic annotation based on GTDB-Tk v2.3.2 showed that these MAGs were distributed across 34 classified phyla and 72 classified genera, with one additional phylum and one additional genus remaining unclassified. The phylum-level classification accuracy was 99.5%, while the genus-level classification accuracy was only 46.1%. Notably, 97.6% of the MAGs were not annotated to the species level, suggesting that Lake Taihu sediments are an important reservoir of microbial dark matter (as shown in Figure 2a). Functional annotation revealed that 57 MAGs (27.7%) possessed complete rRNA-tRNA operons.

**Figure 2:**
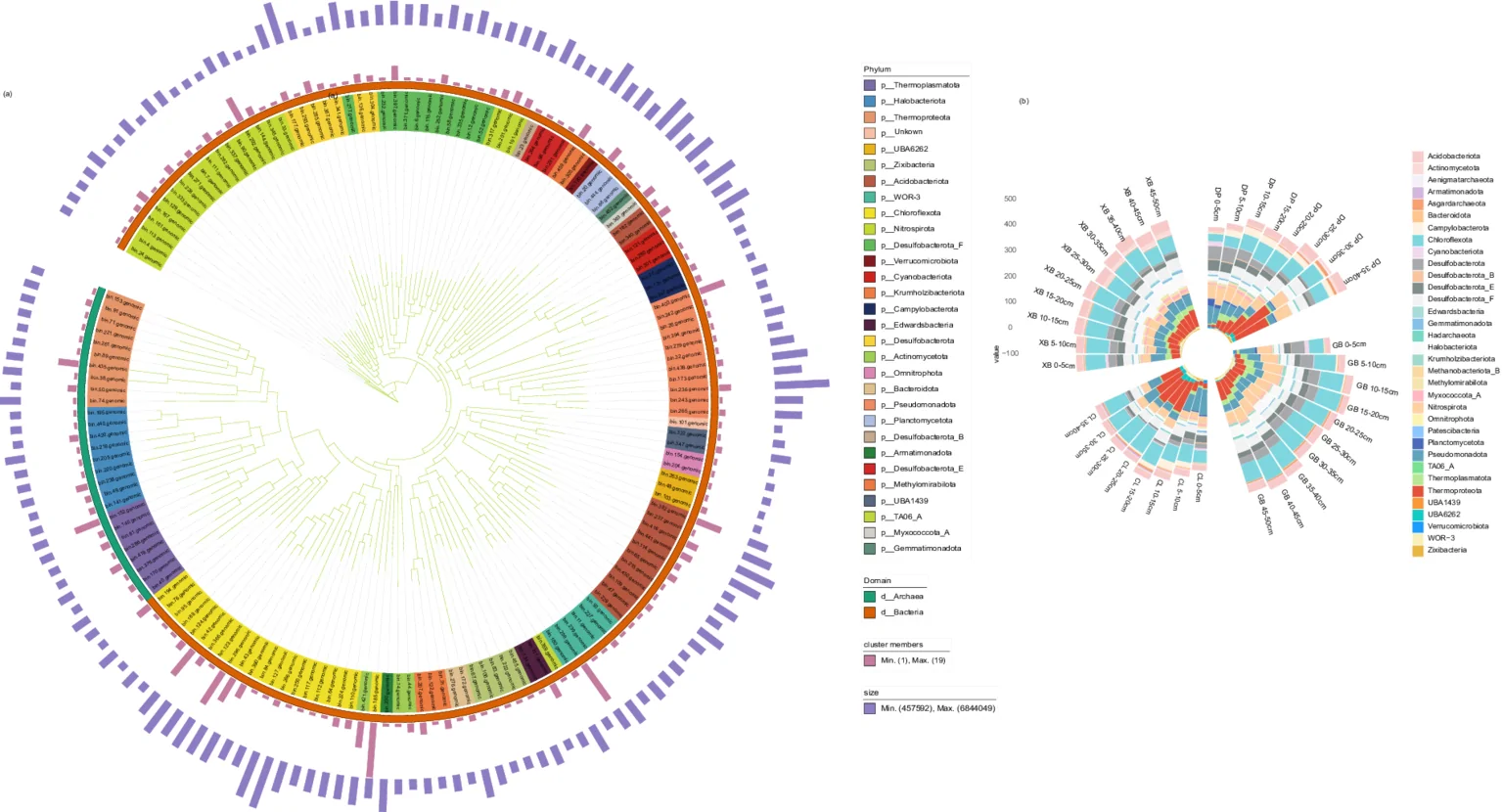
Phylogeny and abundance of 206 dereplicated MAGs. **(a)** Maximum-likelihood phylogenetic tree of 206 dereplicated MAGs. The inner circle represents the domain-level classification, with Archaea and Bacteria distinguished by color. MAG counts per phylum ranged from 1 to 19, and genome sizes ranged from 0.46 Mbp to 6.84 Mbp. **(b)** Aggregated relative abundance of MAGs at the phylum level.

Bacterial MAGs were dominated by Acidobacteriota and Chloroflexota (each 15.7%), while archaeal MAGs were overwhelmingly represented by Thermoproteota (60.6%) (Figure 2b). The most abundant MAGs belonged to the *SpSt-501*, *JAGVSZ01*, and *DSOS01* genera. At the phylum level, Chloroflexota, Thermoproteota, and Pseudomonadota exhibited the highest abundances (17.8, 13.2, and 10.0%, respectively). Cyanobacterial MAGs exhibited a significant vertical stratification pattern, with the highest abundance in the 0–5 cm surface sediment layer (2.27–4.83%) and a sharp decline below 5 cm. Spatial heterogeneity was also observed in the maximum detection depth of cyanobacterial MAGs across lake regions.

### 3.2 Predicted metabolic potential for C-N-S cycling

Functional annotation of the 206 dereplicated MAGs revealed widespread genomic potential for carbon, nitrogen, and sulfur cycling, with notable spatial variation across lake regions (as shown in Figure 3). Genes associated with nitrite ammonification were the most frequently detected (*n* = 44 MAGs), followed by those encoding nitric oxide reduction (*n* = 29), nitrate reduction (*n* = 25), and nitrite oxidation (*n* = 12). Gene coverage for nitrite ammonification in Xu Bay reached 31.7%, suggesting a prominent genomic potential for this pathway in that region. Genes associated with sulfur oxidation were the most frequently detected (*n* = 105 MAGs), followed by those encoding sulfate reduction (*n* = 46) and sulfite oxidation (*n* = 46). Gene coverage for sulfur oxidation varied across regions, ranging from 34.8 to 59.5%. Genes associated with organic carbon oxidation were the most frequently detected (*n* = 205 MAGs), followed by those encoding fermentation (*n* = 170), carbon fixation (*n* = 114), acetate oxidation (*n* = 56), and hydrogen oxidation (*n* = 50). The most common carbon fixation pathway was the Calvin cycle in photosynthesis, in which enzymes such as type I RuBisCO, using energy derived from light or other energy sources, incorporate CO₂ into organic acceptor molecules, which ultimately yield sugars and other organic compounds that support cellular growth and metabolism.

**Figure 3:**
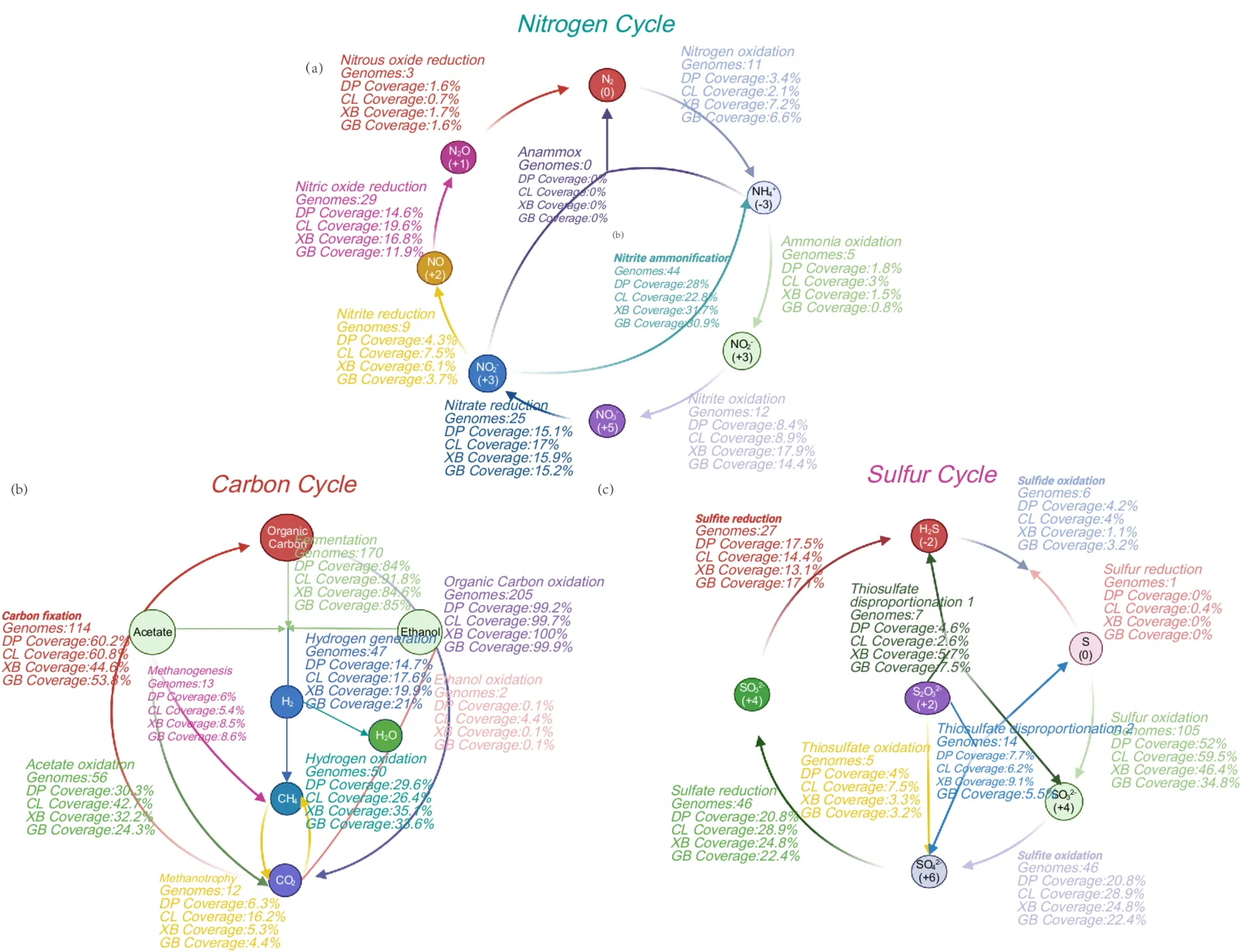
Metagenomic potential and functional gene coverage of nitrogen, carbon, and sulfur cycling pathways. **(a)** Number of MAGs and corresponding gene coverage for each step of the nitrogen cycling pathway; **(b)** Number of MAGs and corresponding gene coverage for each step of the carbon cycling pathway; **(c)** Number of MAGs and corresponding gene coverage for each step of the sulfur cycling pathway. Created in BioRender. Wei, Q. (2026). https://BioRender.com/3a6ystr; https://BioRender.com/mhlcj1x.

Predicted metabolic potential inferred from METABOLIC analysis (Figure 4; Supplementary Figure S1) showed that amino acid utilization had the highest MW score (MW = 7.9), with Chloroflexota contributing 32.5% of the functional potential. Fermentation had the second highest MW score (MW = 7.4), with representatives from 23 phyla and Chloroflexota as the major contributor (33.7%). While Cyanobacteriota contributed substantially to carbon fixation (Rubisco, 22.3%), their contributions to amino acid utilization, complex carbon degradation, and aromatic compound degradation were minimal (0.1–0.6%). Pseudomonadota and Chloroflexota made major contributions to the carbon, nitrogen, and sulfur cycles. Chloroflexota contributed over 50% to 12 metabolic pathways in the carbon, nitrogen, and sulfur cycles, while Pseudomonadota contributed over 50% to 6 pathways.

**Figure 4:**
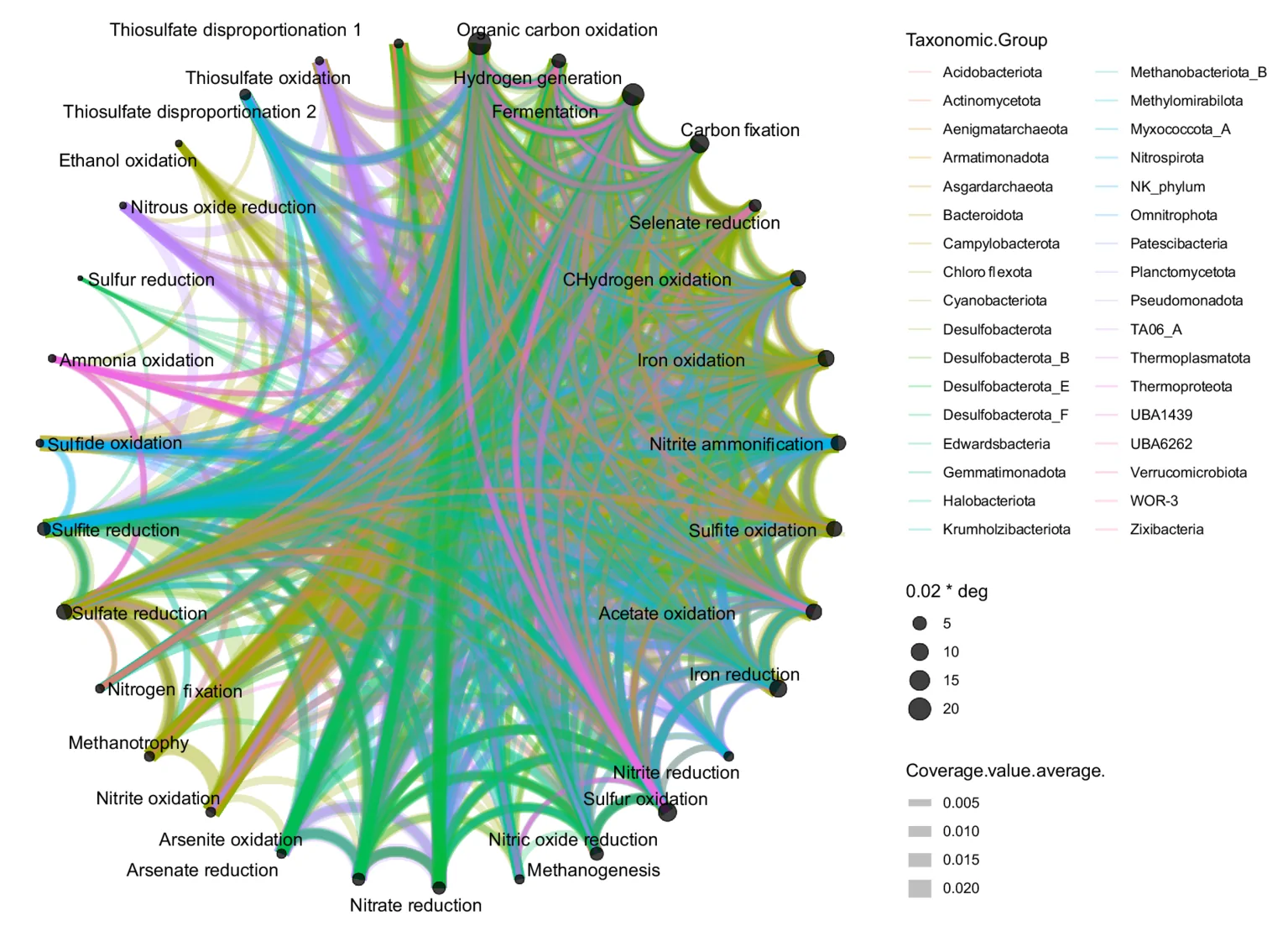
Metabolic functional network constructed from MAG-encoded genes. Node size represents the gene coverage of individual metabolic steps, and edge thickness indicates the average gene coverage between connected steps.

Seven MAGs spanning Nitrospirota, Chloroflexota, Desulfobacterota-F, and Thermoproteota were identified as carriers of genes associated with carbon, nitrogen, and sulfur metabolic modules (Figure 5). Nitrogen cycling genes showed phylum-level differentiation: Thermoproteota (bin.3) harbored *amoA*, while Chloroflexota (bin.42) carried *nrfA/H*, *nirD*, and *nxrA/B*. Sulfur metabolism genes were distributed across multiple phyla: six MAGs carried *sat*; *sdo* was present in Nitrospirota (bin.228), Thermoproteota (bin.3), and Desulfobacterota-F (bin.397); and *phsA* was present in bin.397. The Chloroflexota MAG (bin.250) simultaneously carried *sat*, *nirK*, and *norC* genes. Furthermore, four putative sulfate-reducing MAGs containing the *dsrA* gene were also found. The distribution of these genes suggests a genomic potential for metabolic versatility among sediment microbial communities, though their actual functional activity and ecological relevance require further validation through metatranscriptomic or biochemical approaches.

**Figure 5:**
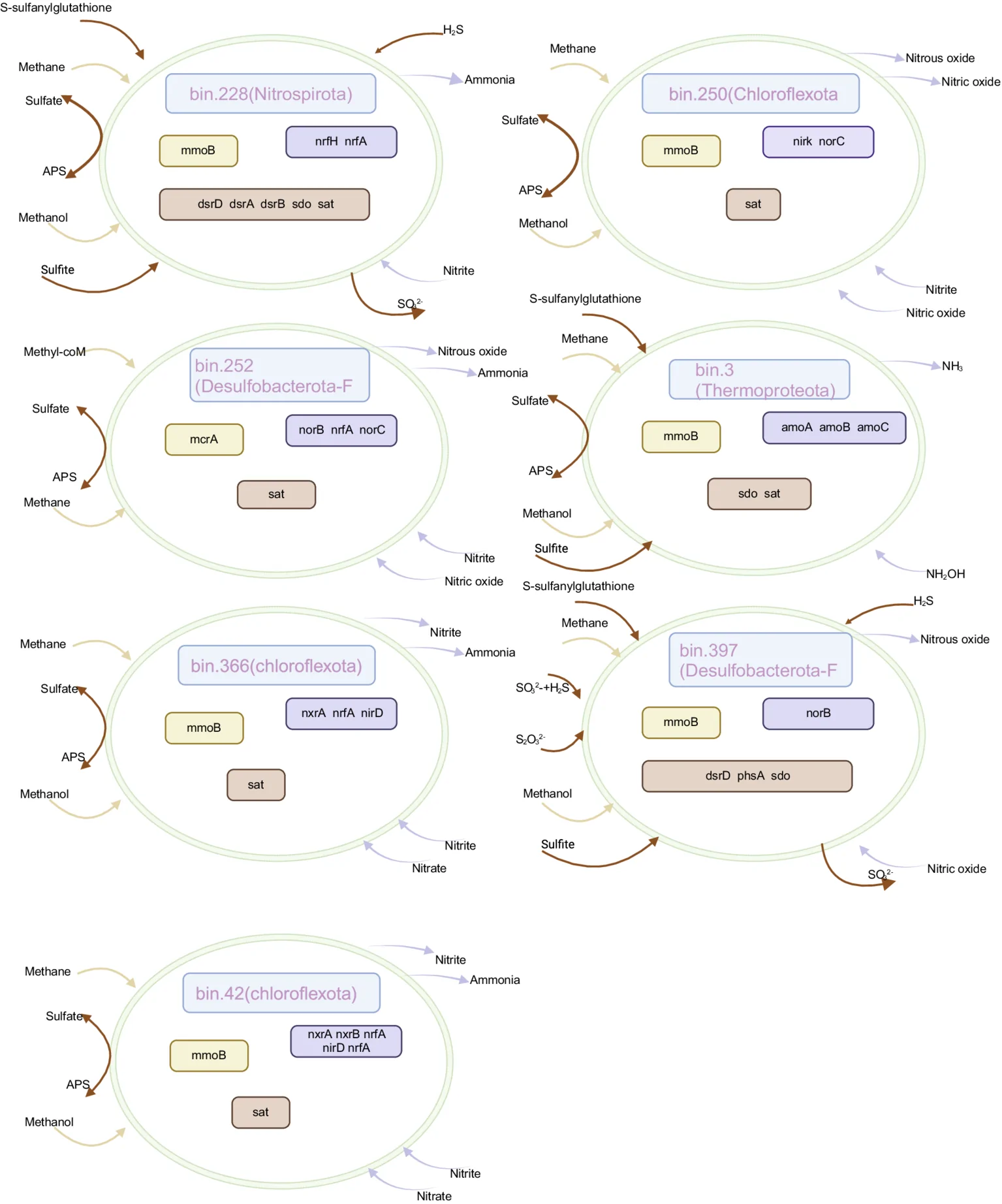
Cross-domain coupling of CH_4_, N, and S cycles in MAGs. Created in BioRender. Wei, Q. (2026). https://BioRender.com/8jtwbhb.

Three cyanobacterial MAGs were recovered in this study and taxonomically assigned to *Microcystis wesenbergii* (bin.121) and M. *panniformis* (bin.280, bin.301). Gene sets for assimilatory nitrate reduction (*nasA, nirA*), sulfate reduction (*cysC, cysH*), and the Calvin cycle were identified in these MAGs, suggesting genomic capacity for utilizing nitrate and sulfate as nutrient sources and for CO_2_ fixation (Figure 6). Notably, although the cyanobacterial MAGs lacked nitrogen fixation genes, they encoded assimilatory nitrate reductase (EC 1.7.7.1) and nitrite reductase (EC 1.7.7.2). Furthermore, phosphorus serves as a key element limiting phytoplankton growth, and multiple phosphorus uptake-related gene clusters (e.g., pstSCAB) were also detected in the MAG. Photosynthetic electron transport components, including the cytochrome b_6_ complex and F-type ATP synthase, were also present. The bin.280 genome harbored multiple CRISPR-Cas gene types, comprising type II-D (Cas9), type III-B (Cmr complex), and type I-D (Csc complex). The type II-D system may provide defense against DNA bacteriophages, the III-B system may target RNA viruses, and the I-D system may offer broad-spectrum defense against mixed infections. The ATP-binding cassette (ABC) transporter repertoire identified in the cyanobacterial MAGs included systems for amino acid uptake (*aapJ/Q*), iron acquisition (*ABC. FE*), manganese transport (*ABC. MN*), phosphate uptake (*ABC. PA*), heavy metal detoxification (*ABC. CD*), oligopeptide uptake (*ABC. PE*), and efflux of metabolic waste and antibiotics (*ABC2*). Collectively, these genomic features suggest that cyanobacteria in Lake Taihu sediments possess a broad genomic arsenal for nutrient competition, stress tolerance, and energy metabolism, although their actual expression and ecological roles await functional validation.

**Figure 6:**
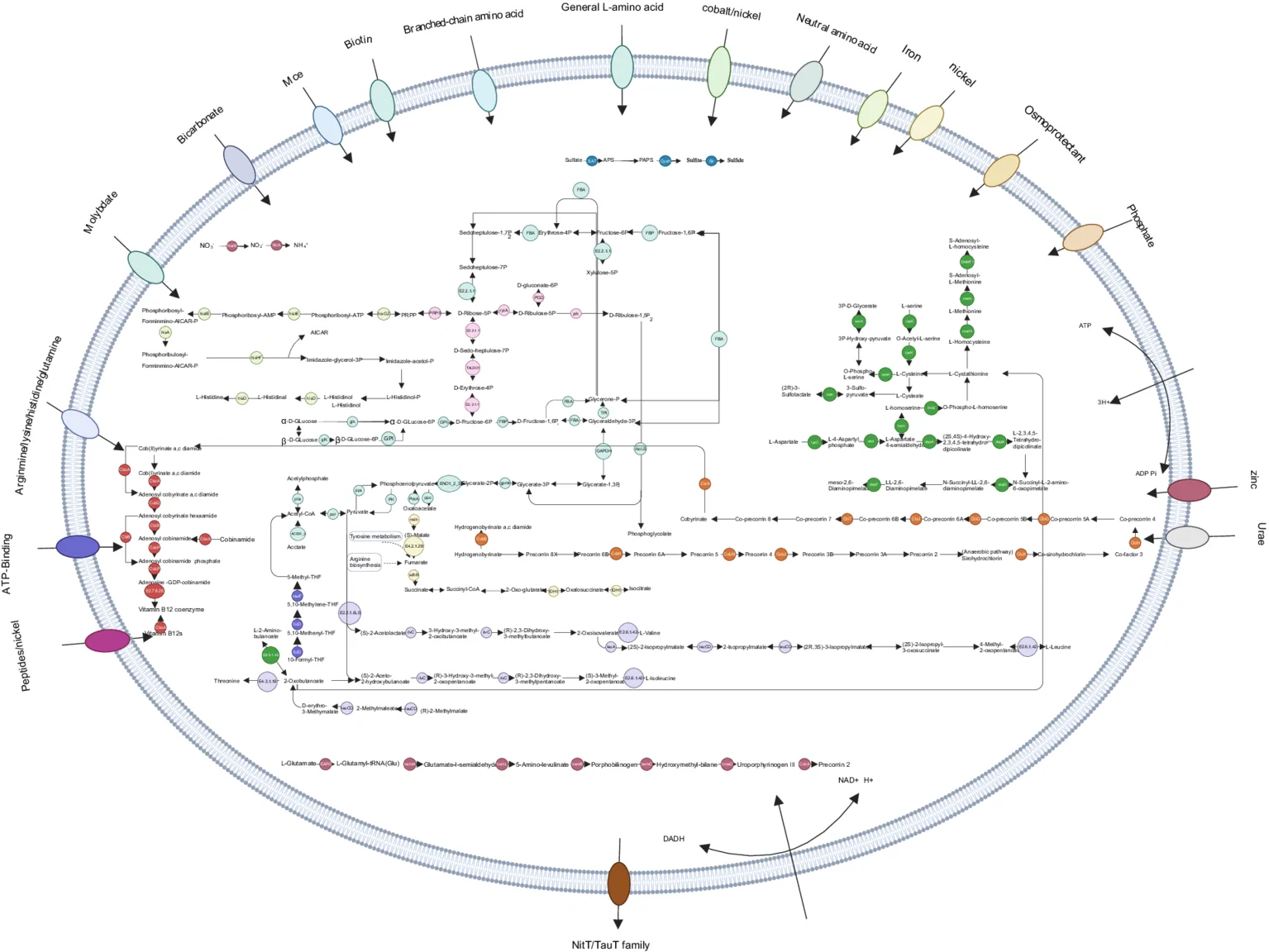
Visualization of cyanobacterial MAG metabolic pathways. Created in BioRender. Wei, Q. (2026). https://BioRender.com/ok5v113.

### 3.3 Secondary metabolite biosynthetic potential and Pangenomic features

Analysis of 206 dereplicated MAGs identified 295 secondary metabolite biosynthetic gene clusters, with 55.3% of MAGs (*n* = 114) containing at least one BGC. The number of BGCs per genome ranged from 1 to 16, with the highest count observed in a Pseudomonadota MAG (*Achromobacter insuavis_A*). BiG-SCAPE clustering classified these BGCs into eight functional categories, dominated by ribosomally synthesized and post-translationally modified peptides (RiPPs, 29.2%), non-ribosomal peptide synthetase (NRPS) gene clusters (23.4%), and terpenoid BGCs (21.7%) (Figure 7).

**Figure 7:**
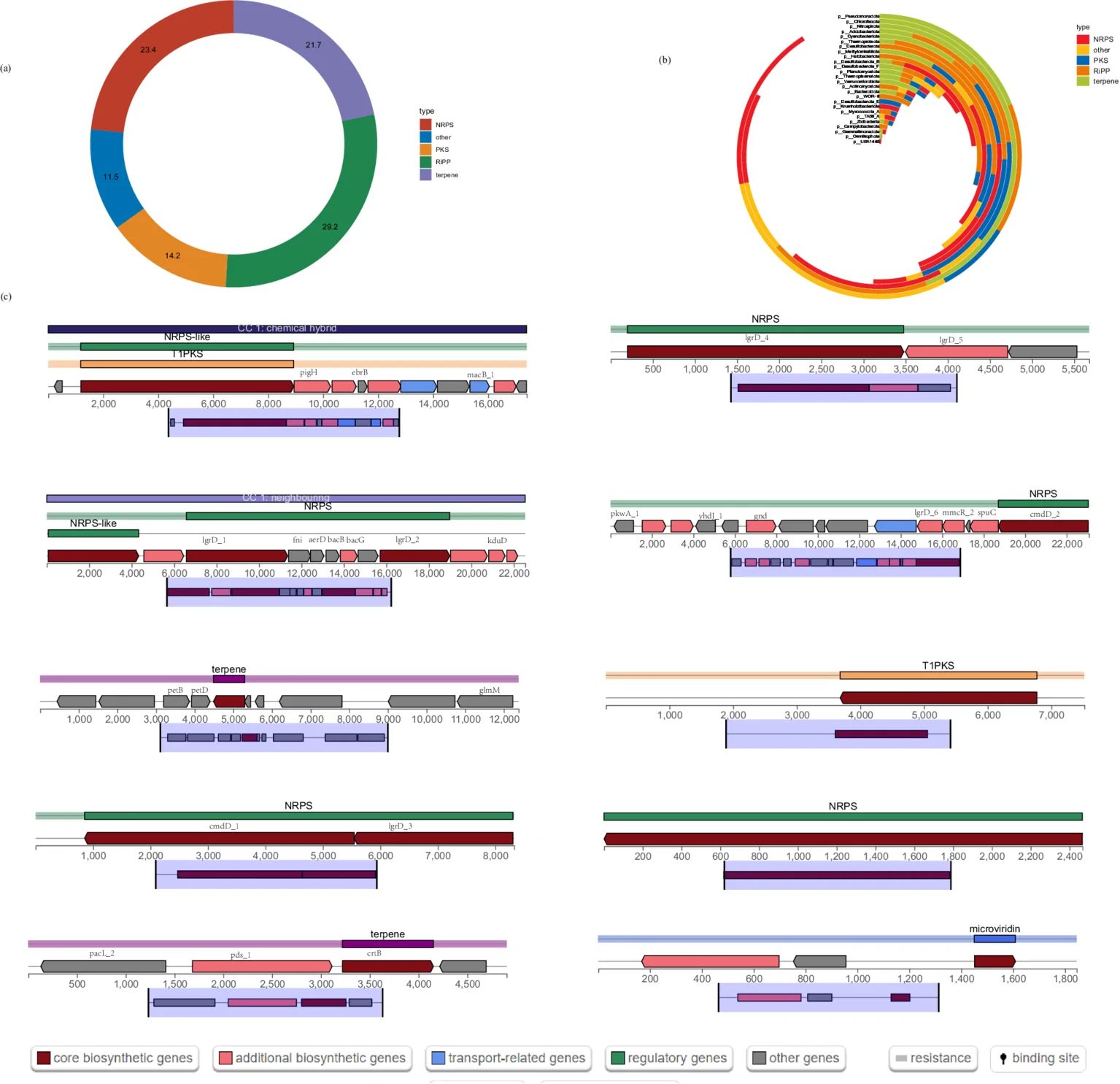
Analysis of natural secondary metabolite BGCs in MAGs. **(a)** Taxonomic classification of BGCs detected in all MAGs. **(b)** Phylum-level distribution of BGC abundance and diversity. **(c)** Detailed BGC composition of cyanobacterial MAGs.

Phylum-level biosynthetic capacity was non-randomly distributed. Pseudomonadota exhibited substantial metabolic versatility, carrying 9 terpenoid BGCs, 9 NRPS gene clusters, and 14 “Other-type” BGCs, which may facilitate adaptation to environmental stress. Nitrospirota carried 9 NRPS gene clusters, 6 terpenoid BGCs, and 6 polyketide synthase (PKS) gene clusters. Chloroflexota emerged as a major secondary metabolite reservoir, harboring 10 NRPS gene clusters, 5 terpenoid BGCs, 4 PKS gene clusters, and 2 RiPPs. The NRPS gene clusters include genes potentially involved in toxin synthesis, the terpenoid clusters contain genes for carotenoid biosynthesis, and the PKS gene clusters may contribute to secondary metabolite production. In addition, genes such as *cmdD* (aromatic compound degradation), *lgrD* (lipopolysaccharide synthesis), *pigH* (phycobilisome stability), *pds_1* (carotenoid synthesis), *spvc* (cyanobactin regulation), and *mmcR2* (polyphosphate metabolism) were also identified, suggesting a broad genomic capacity for environmental adaptation. However, these functional predictions are based on gene content alone and require experimental validation.

### 3.4 Resistome, CRISPR–Cas defense, and predicted HGT networks

Resistome profiling of the 206 dereplicated MAGs using RGI v6.0.3 identified 101 antibiotic resistance genes (Figure 8). The resistance landscape was dominated by glycopeptide resistance determinants, with *vanT* accounting for 59.4% and *vanW* for 15.8% of all ARGs (75.2% combined), followed by resistance-nodulation-division (RND)-type efflux pumps (10.9%), which are predicted to be associated with resistance to multiple antibiotic classes, including tetracyclines, fluoroquinolones, and macrolides. Within the glycopeptide resistance category, *vanT* was distributed across multiple phyla, including Chloroflexota, Cyanobacteriota, Nitrospirota, Desulfobacterota-F, and Acidobacteriota. The *vanW* gene was predominantly found in Chloroflexota, whereas *vanY* and *vanH* occurred sporadically in Cyanobacteriota and Planctomycetota, respectively. Notably, bin.280 (*Microcystis panniformis*) harbored both *vanT* and *vanY* alongside NRPS/PKS BGCs, and bin.236 (*Achromobacter insuavis_A*) carried an array of RND pumps, an OXA β-lactamase, and several BGCs for NRPS, terpenes, and hydrogen cyanide.

**Figure 8:**
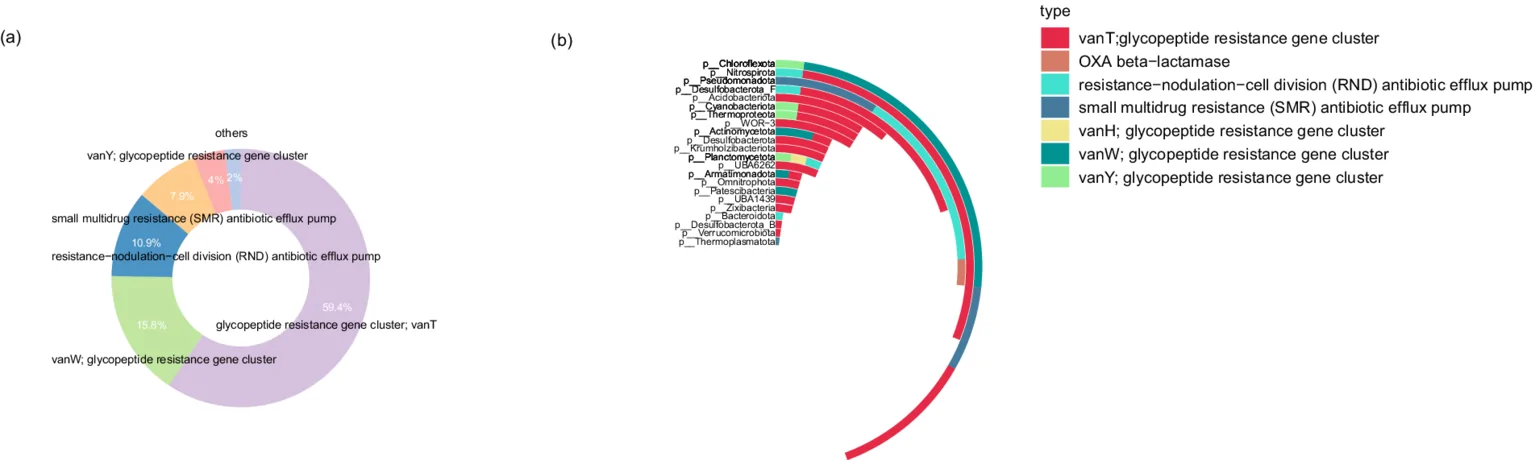
Distribution of putative ARG types across Lake Taihu sediment MAGs. **(a)** ARG classification; **(b)** ARG distribution across phyla.

CRISPR-Cas loci were identified in 13 of the 206 MAGs, which were phylogenetically distributed across eight phyla (Figure 9). The majority of the CRISPR-positive MAGs belonged to Chloroflexota (bins 110, 250, 266, and 64) and Cyanobacteriota (bins 121, 280, and 301). The remaining six MAGs were affiliated with Thermoproteota (bin.91), Desulfobacterota_F (bin.217), WOR-3 (bin.227), Armatimonadota (bin.230), Acidobacteriota (bin.237), and Desulfobacterota (bin.293). Following a strict classification based on the completeness of both the Adaptation and Interference modules extracted from the cas_operons_putative predictions, only two MAGs (bin.217 and bin.227) harbored complete CRISPR-Cas systems, with both modules achieving 100% completeness. In contrast, the remaining 11 MAGs possessed partial systems, predominantly characterized by absent or incomplete adaptation modules. Notably, MAG bin.280 encodes three coexisting CRISPR-Cas types (II-D, III-B, and I-D); however, all three systems were classified as partial due to incomplete adaptation components. Phylum-level analysis further revealed that the WOR-3-associated MAG bin.227 represents the sole genome within its phylum to carry a Type I-C system. The occurrence of CRISPR-Cas systems in the sediment metagenomes is potentially associated with local phage predation pressures, although their actual protective efficacy requires further functional validation.

**Figure 9:**
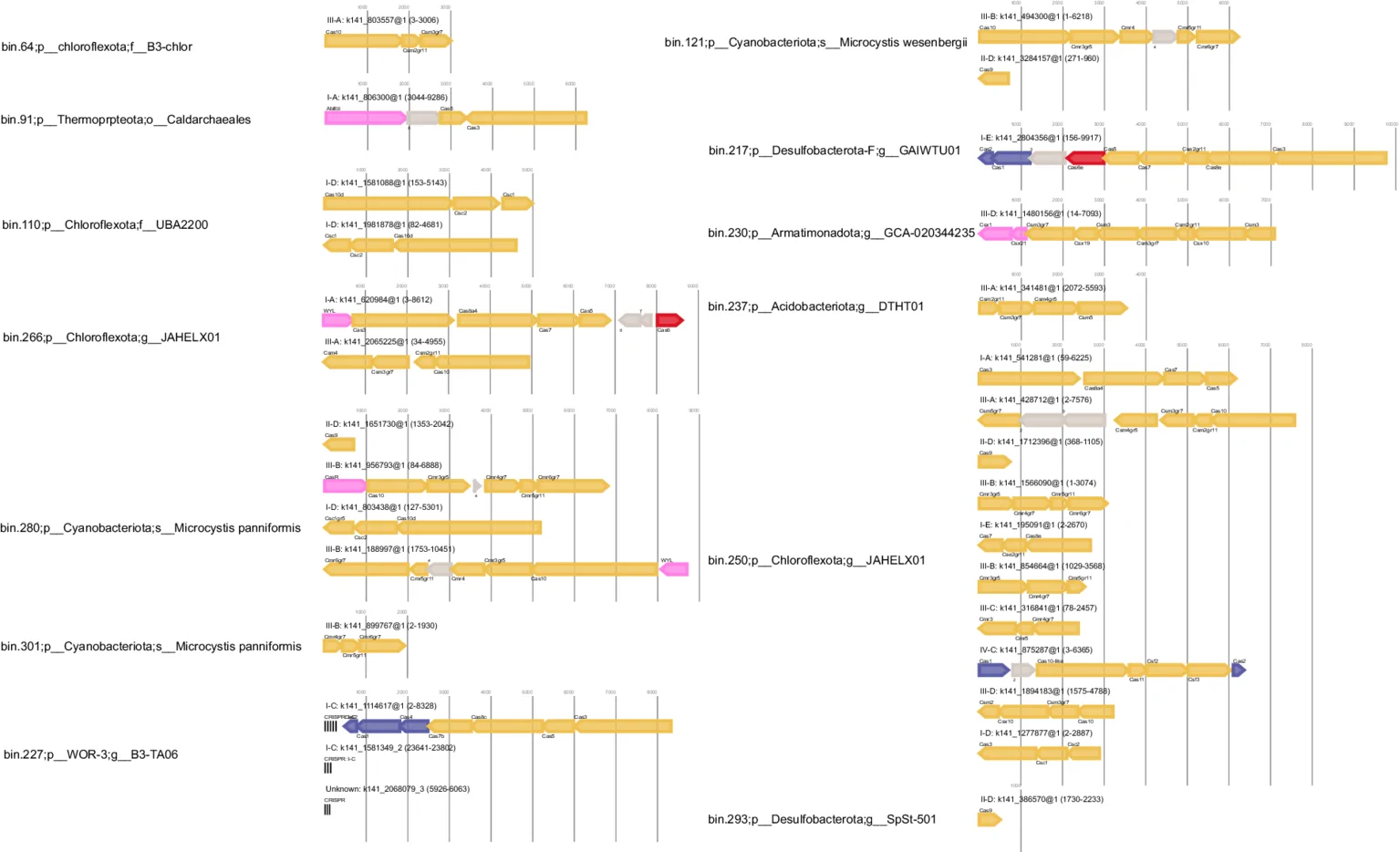
CRISPR-Cas systems and arrays detected by CRISPR-CasTyper.

Integration of MAG-based evolutionary genomics suggested extensive predicted horizontal gene transfer networks within the sediment microbiome. A total of 490 predicted HGT events with defined directionality were identified across all MAGs (Figure 10), of which 18.8% (*n* = 92) exhibited recent transfer characteristics (genetic divergence <1%). A total of 86.4% of MAGs participated in at least one predicted HGT event, with donor and recipient coverage of 61.7 and 71.8%. Notably, 42.2% of MAGs were identified as candidates for recent predicted HGT events, with donor and recipient participation rates of 24.8 and 28.2%, suggesting that environmental pressures may contribute to the predicted reshaping of the gene pool via selective forces. However, these HGT predictions are inferred from genomic data and should be considered candidate events requiring further experimental or metatranscriptomic validation before they can be confirmed as bona fide transfer events.

**Figure 10:**
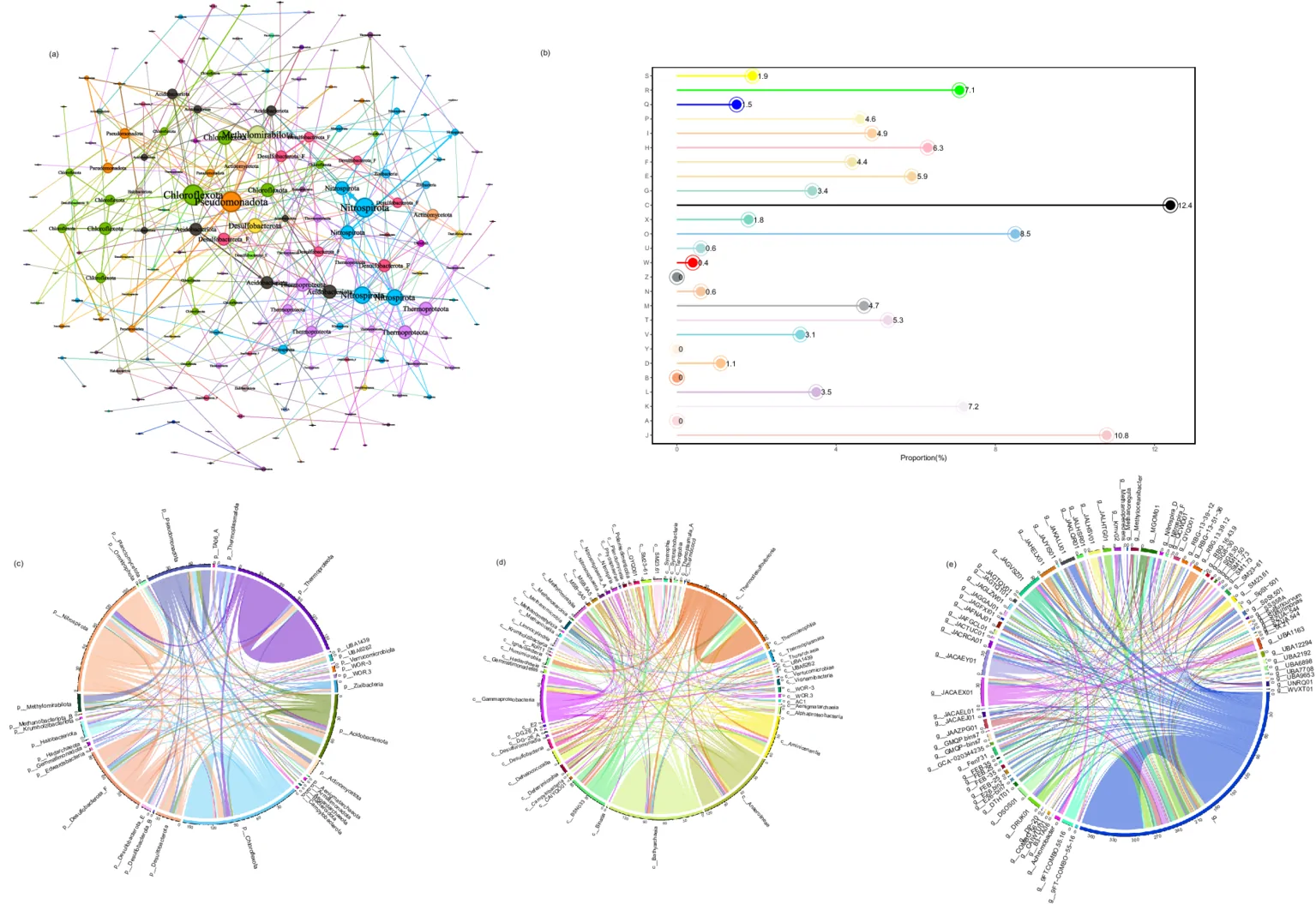
Horizontal gene transfer (HGT) network and its functional and taxonomic characteristics. **(a)** Directed HGT network among MAGs. Nodes colored by phylum; edge width denotes transfer frequency; **(b)** COG functional categories of HGT-flanking genes. Shown are significantly enriched categories; **(c–e)** HGT event frequencies summarized at **(c)** phylum, **(d)** class, and **(e)** genus levels.

Among the predicted HGT candidates identified in MAGs, the dominant participating phyla included Nitrospirota, Chloroflexota, and Thermoproteota, suggesting a possible association between predicted HGT frequency and phylogenetic lineage. Clusters of Orthologous Groups (COG) functional annotation of transferred genes revealed enrichment in three core functional modules: energy metabolism (12.4%), translation machinery (10.8%), and transcriptional regulation (7.2%), which together accounted for 30.4% of all predicted HGT candidates. These enriched categories may reflect genomic traits that are frequently transferred among lineages, potentially contributing to metabolic versatility.

Additionally, 24 ARG transfer events were predicted, predominantly distributed among Chloroflexota, Nitrospirota, Acidobacteriota, Desulfobacterota_F, Thermoproteota, Desulfobacterota, Desulfobacterota_E, and Pseudomonadota. The co-occurrence of these predicted ARG transfer events with the high prevalence of glycopeptide resistance genes (75.2% of the resistome) suggests a potential for resistance gene dissemination under eutrophication pressure, although this remains speculative and requires further investigation through genomic-context analysis and functional assays.

## 4 Discussion

### 4.1 Microbial community metabolic networks and coupled carbon-nitrogen-sulfur cycling: insights from genomic potential

Through multidepth metagenomic analysis of Lake Taihu sediments, we obtained 206 dereplicated MAGs, revealing a genomic potential for metabolic versatility in the freshwater sediment microbial community. Analysis of metagenome-assembled genomes suggested that microorganisms may play multiple functional roles with potential interactions in carbon, nitrogen, and sulfur cycling, thereby contributing to the balance and cycling of elements within the ecosystem. Notably, the Chloroflexota MAG (bin.250) simultaneously carried *sat, nirK*, and *norC* genes, suggesting composite metabolic potential for participating in both sulfur cycling and denitrification processes.Furthermore, four MAGs containing the putative dsrA gene were also found to harbor a subset of genes involved in denitrification and dissimilatory nitrate reduction to ammonium (DNRA). The presence of either DNRA or denitrification capabilities appears to be a feature among sulfate-reducing bacteria, indicating their metabolic flexibility in response to electron acceptor availability (Baker et al., 2015). A MAG from a filamentous microorganism (taxonomically assigned to the phylum Proteobacteria, class Gammaproteobacteria, family Beggiatoaceae) has been shown to be involved in multiple metabolic processes, including denitrification, DNRA, and thiosulfate oxidation (Milucka et al., 2012). Genomic analysis revealed the presence of *narG/H* genes along with *nxrA/B* genes, implicating its potential role in the oxidation of nitrite (NO_₂_^−^) in sedimentary environments (Zhang et al., 2024a). Furthermore, analysis of mangrove sediments revealed a coupled phosphorus-nitrogen metabolic mechanism, in which MAGs assimilated NH₄^+^ into biomass nitrogen through *glnA*-mediated glutamine synthesis, thereby potentially contributing to organic phosphorus mineralization and facilitating tightly coupled nitrogen-phosphorus metabolism (Liu et al., 2025). Such MAGs with cross-cycle gene content may facilitate their adaptation to environmental stresses (Lin et al., 2021).

### 4.2 Genomic features of cyanobacterial MAGs

Three cyanobacterial MAGs were recovered in this study and taxonomically assigned to *Microcystis wesenbergii* (bin.121) and *M. panniformis* (bin.280, bin.301). We described the genomic features identified in these MAGs and contextualized them against published cyanobacterial reference genomes.

Our findings suggest that the carbon fixation pathway in the cyanobacterial phylum was based on Form I RuBisCO. The Calvin-Benson-Bassham (CBB) cycle is recognized as the principal mechanism for biological inorganic carbon fixation on Earth. To date, all studied cyanobacterial lineages have been found to encode carbon fixation mechanisms dependent on Form I RuBisCO within CO₂-concentrating carboxysomes (Jaffe et al., 2024). Notably, although the cyanobacterial MAGs analyzed in this study lacked nitrogen fixation genes, they encoded assimilatory nitrate reductase (EC 1.7.7.1) and nitrite reductase (EC 1.7.7.2). When available nitrogen sources are present, these nitrate and nitrite reductase genes facilitate the assimilation of environmental nitrate/nitrite into ammonium (Alcorta et al., 2020). In cyanobacteria, nitrate uptake and the sequential reduction of nitrate to nitrite and ammonium are supported by ATP and reduced ferredoxin generated from photosynthetic electron transport. Following uptake via high-affinity ABC-type transporters or major facilitator superfamily (MFS) permeases, the imported nitrate/nitrite is sequentially reduced to nitrite and ammonium through ferredoxin-dependent metalloenzymes (Flores et al., 2005).

Phosphorus serves as a key element limiting phytoplankton growth, and the detection of multiple phosphorus uptake-related gene clusters (e.g., *pstSCAB, sphX, phoA/phoD/phoX*) in cyanobacteria indicates their capacity to employ diverse mechanisms to fulfill phosphorus acquisition requirements across varying environmental conditions (Shimura et al., 2021). Our results are consistent with this notion, as we have obtained similar findings. For sulfur assimilation, genes encoding sulfate kinase (*cysC*) and phosphoadenosine phosphosulfate reductase (*cysH*) were present in the MAGs. These enzymes catalyze the conversion of activated sulfate to sulfite, a conserved step in the assimilatory sulfate reduction pathway that has been documented across cyanobacteria, bacteria, fungi, and plants (Frangeul et al., 2008).

We compared our MAGs against published cyanobacterial genomes. Previous comparative genomic studies have established that approximately 559 genes constitute the core genome conserved across 58 cyanobacterial strains representing six different orders (Simm et al., 2015). This core genome includes genes encoding RuBisCO and other central metabolic functions. Similarly, assimilatory nitrate reduction (*narB*, *nirA*) and sulfate assimilation (*cysC*, *cysH*) genes have been documented in diverse cyanobacterial genomes, including those of *Microcystis*, *Synechococcus*, and *Nostoc* lineages (Bird and Wyman, 2003). These observations suggest that the metabolic features detected in our MAGs largely reflect conserved cyanobacterial core-genome properties rather than sediment-specific adaptations.

Genes encoding CRISPR–Cas components were identified in bin.280, including type II-D (Cas9), type III-B, and type I-D. The coexistence of multiple CRISPR–Cas subtypes within a single genome is consistent with previous findings. A study showed that each of the 14 *Microcystis aeruginosa* genomes analyzed contained at least one CRISPR locus, with subtypes I-A, I-D, III-A, and III-B identified across the strains (Yang et al., 2015). Furthermore, in a natural population of *Microcystis aeruginosa* from a small eutrophic pond, 52 out of 94 isolates carried CRISPR arrays, which were classified into 22 distinct CRISPR-associated genotypes (Kuno et al., 2014). The presence of CRISPR-Cas loci in several of our MAGs suggests a potential role in phage defense and genomic stability, consistent with observations in marine cyanobacteria. However, functional confirmation would require experimental validation of spacer target interactions.

The cyanobacterial MAGs encoded a diverse repertoire of ABC transporters, including systems predicted to function in amino acid uptake (*aapJ/Q*), iron acquisition (*ABC. FE*), manganese transport (*ABC. MN*), phosphate uptake (*ABC. PA*), heavy metal detoxification (*ABC. CD*), oligopeptide uptake (*ABC. PE*), and efflux of metabolic waste and antibiotics (*ABC.2*). ABC transporters have been reported to be represented in cyanobacterial pangenomes. Driscoll et al. (2018) found that ABC-type transporters are among the most highly represented gene categories in the pangenome of a globally distributed clade of bloom-forming cyanobacteria within the Nostocales. The phosphate uptake system (PstSCAB) detected in our MAGs aligns with previous genomic observations in cyanobacteria, in which the phosphate-binding protein PstS binds extracellular inorganic phosphate and transfers it to the transmembrane proteins PstC and PstA, followed by ATP hydrolysis-dependent translocation via PstB (Caille et al., 2024). Beyond nutrient uptake, ABC efflux pumps have been associated with multidrug tolerance and heavy metal resistance in cyanobacteria and coexisting bacteria (Zhang et al., 2020).

Notably, given the limited number of cyanobacterial MAGs recovered in this study (*n* = 3) and their moderate quality, these findings are preliminary and require further validation through additional genome recovery and functional assays.

### 4.3 Biosynthetic gene clusters of natural secondary metabolites and their relevance to cyanobacteria

In this study, we employed AntiSMASH v7.1 coupled with the MIBiG v2.0 database of known gene clusters to identify putative BGCs across 206 dereplicated MAGs, revealing a total of 295 putative BGCs. Among the identified BGC types, RiPPs, non-ribosomal peptide synthetase products (NRPS), polyketide synthase products (PKS), and terpenes constituted the predominant categories. This observation aligns with established research indicating that RiPPs occur more frequently in terrestrial systems, whereas non-ribosomal peptides (NRPs), polyketides (PKs), and terpene BGCs are widely distributed across multiple ecosystems (Yi et al., 2024). At the phylum level, Chloroflexota exhibited the most prolific secondary metabolic potential, encoding diverse biosynthetic pathways including terpenes, RiPPs, and non-ribosomal peptide synthetase (NRPS) products. Terpenes and their modified derivatives have been reported to exhibit notable antibacterial, antifungal, and anthelmintic activities, primarily through disrupting the structural integrity of pathogenic microorganisms and interfering with their metabolic processes, thereby potentially conferring significant defensive capabilities (Masyita et al., 2022). RiPPs represent a class of peptide-derived natural products exhibiting diverse pharmacological activities. These compounds are synthesized as ribosomal peptides and subsequently undergo leader peptide-directed enzymatic modifications, endowing them with potent antibiotic, antiviral, and antitumor functions (Walker et al., 2022). Our findings align with existing literature, as lineages including Chloroflexota, Planctomycetota, and certain members of Pseudomonadota are similarly reported to harbor abundant BGCs, suggesting considerable biosynthetic potential (Wang et al., 2024). Pseudomonadota establishes chemical warfare dominance through large-scale synthesis of siderophores, broad-spectrum antibiotics, and complex polyketide toxins, while concurrently employing RiPPs and terpenoid signaling molecules for microenvironment modulation (Zhang et al., 2023).

As cyanobacteria are one of the primary sources of secondary metabolites, their genomes are enriched with modular NRPS and PKS pathways. Secondary metabolites such as pyocyanin, cyanopeptolin, and microcystin may provide ecological advantages to *Microcystis* by conferring resistance against grazers, mediating allelopathic interactions, facilitating quorum sensing, and/or enhancing protection from reactive oxygen species (ROS), thereby potentially establishing competitive dominance in aquatic environments (Kehr et al., 2011). Cyanobacteria in high-temperature hot springs exhibit a significantly lower abundance of BGCs than those in non-thermal environments such as lakes, consistent with their adaptive modulation of secondary metabolite production in response to distinct ecological pressures (Alcorta et al., 2020). In response to persistent extreme stresses, Antarctic cyanobacteria produce secondary metabolites such as mycosporine-like amino acids (MAAs) with UV-protective and anti-inflammatory activities, suggesting significant biotechnological potential (Freitas et al., 2024). The expression of BGCs is likely downregulated due to diminished ribosome biogenesis and energy supply, thereby limiting the synthesis of secondary metabolites. Although exogenous nitrogen sustains survival, it fails to effectively reactivate BGC expression under phosphorus deprivation (Wei et al., 2023).

### 4.4 Horizontal gene transfer and dissemination of antibiotic resistance genes

ARGs are ubiquitously distributed in aquatic environments, where their dissemination may be associated with anthropogenic activities and aquatic environmental characteristics. Through mechanisms including HGT, ARGs propagate within microbial communities, resulting in extensive contamination with complex transmission pathways. The potential transfer of these genes to commensal bacteria and pathogens remains a concern that warrants continued surveillance (Li et al., 2025).

In the present study, the *vanT* gene cluster represented the predominant resistance determinant, accounting for 59.4% of all identified putative antibiotic resistance genes, followed by the *vanW* gene cluster and RND-type antibiotic efflux pumps. Consistent with our observations, a previous study (Zhang et al., 2024b) detected 18 subtypes of antibiotic resistance genes in sediment subsamples from Lake Taihu. Efflux pump genes contributed to more than 49% of the total resistance gene abundance. In contrast, ARG types and distributions exhibit distinct profiles across other ecosystems. For instance, seawater environments are enriched with peptide and multidrug resistance genes, while rifamycin and β-lactam resistance genes dominate marine sediment habitats. In algal-bacterial symbiotic systems, Proteobacteria, Cyanobacteria, Bacteroidota, and Actinobacteriota are the main potential hosts of ARGs, which differs from our results. Our findings indicate that Chloroflexota, Nitrospirota, and Desulfobacterota_F are the primary potential host phyla (You et al., 2024). The distribution and dissemination of putative ARGs are influenced by multiple factors. Cyanobacterial blooms have been reported to serve as a potential factor for the spread and enrichment of ARGs in freshwater systems. *Microcystis aeruginosa* and *Planktothrix* may facilitate this process by creating diverse micro-niches through phycosphere formation, enhancing bacterial diversity, and promoting the enrichment of antibiotic-resistant bacteria along with the dissemination of ARGs (Zhang et al., 2020). The abundance and diversity of ARGs in Lake Taihu increase significantly with sediment depth. This has been attributed to the more intense resource competition among functional microorganisms in deeper sediment layers, where carrying ARGs enhances their resistance (Zhang et al., 2024b). Sulfate and nitrate are important factors influencing the distribution of ARGs. Nitrate addition contributes to increased ARG abundance. The impact of environmental factors on ARGs is mainly mediated through indirect effects on bacteria. Nitrate-reducing bacteria or sulfate-reducing bacteria simultaneously carry ARGs, and sulfate-reducing bacteria can expel antibiotics from their cells through efflux pump genes (Jia et al., 2018).

Horizontal gene transfer serves as a recognized force driving microbial evolution and functional adaptation. Our results indicate that the core functional modules involved in predicted HGT events are primarily focused on energy metabolism (12.4%), translation machinery (10.8%), and transcriptional regulation (7.2%). Approximately 34% of horizontally transferred genes in cyanobacteria exhibit uniqueness, being derived from diverse cyanobacterial lineages and participating in replication, metabolism, and signal transduction. Accessory genes showed a notable proportion of HGT-derived sequences (56.4%), which may contribute to energy metabolism and environmental adaptation. Notably, HGT events were also detected in 73 core genes, suggesting their critical role in enhancing metabolic potential and defensive capabilities (Cao et al., 2022). A previous study revealed that the HGT process in composting systems is associated with the combined effects of microbial physiological status, environmental conditions, pollutant influences, and quorum sensing (QS) mechanisms (Huang et al., 2021). Micro-electrolysis also enhances the conjugative transfer of plasmid-mediated antibiotic resistance genes at the microbial community level (Liang et al., 2025).

Multiple studies have suggested that multiple environmental factors can influence the horizontal transfer of ARGs in aquatic environments. This observation aligns with previous research indicating that mobile genetic elements (MGEs) serve as key determinants in shaping the distribution of ARGs. Although we did not measure heavy metals or emerging contaminants in this study, previous studies have documented their presence in Taihu sediments (Wu et al., 2023). The potential contribution of such co-stressors to ARG dissemination in this system remains an open question that warrants integrated physicochemical and metagenomic investigations in future work. Micro-electrolysis significantly accelerates the dissemination of antibiotic resistance genes through conjugation—a key mechanism of horizontal gene transfer—and increases the transfer frequency of plasmids carrying ARGs (Liang et al., 2025). The combined effects of nitrogen and plasmid exposure promote the intra- and extracellular transfer of ARGs in phytoplankton-algal environments. Moderate nitrogen concentrations promote the transfer of ARGs to intracellular DNA of algae-attached microorganisms, extracellular DNA associated with algal cells, and intracellular DNA of free-living microorganisms. In contrast, high nitrogen concentrations primarily facilitate ARG transfer to intracellular DNA of free-living microorganisms. Additionally, large plasmids enhance ARG transfer to extracellular DNA, while small plasmids favor intracellular DNA transfer (Zhang et al., 2020).

All functional interpretations presented here are based on DNA-level metagenomic data and thus reflect genomic potential rather than confirmed *in situ* metabolic activities.

## 5 Conclusion

This genome-resolved study of Lake Taihu sediments reconstructed 206 dereplicated metagenome-assembled genomes spanning 34 classified phyla and 72 classified genera, with 97.6% unclassified at the species level, suggesting extensive microbial dark matter. The community was dominated by bacterial Acidobacteriota and Chloroflexota, and archaeal Thermoproteota. Our genomic analyses suggest potential metabolic coupling among carbon-nitrogen-sulfur cycles, with Chloroflexota and Pseudomonadota emerging as putative metabolic hubs based on gene content. Cyanobacteria possess CRISPR-Cas loci (types II-D, III-B, and I-D), along with ABC transporters and genes involved in C-N-S networks, which may collectively contribute to environmental adaptation; however, the completeness and functionality of these CRISPR systems remain to be validated. Of 295 secondary metabolite biosynthetic gene clusters, 87.6% originated from uncultivated lineages, extending microbial dark matter to chemical dark matter. Putative *vanT/vanW* were the most prevalent ARG-like hits (75.2%). We also identified 490 predicted horizontal gene transfer candidates, of which 18.8% are classified as recent transfers, and these may play a role in shaping genomic diversity. Overall, this study offers a genomic framework for understanding microbial resilience in eutrophic sediments and provides hypotheses that may inform future bloom mitigation and bioremediation efforts upon experimental testing.

## Data Availability

The datasets presented in this study can be found in online repositories. The names of the repository/repositories and accession number(s) can be found in the article/Supplementary material.

## Footnotes

1
https://github.com/songweizhi/MetaCHIP
