# Supplementary material for: A metagenomic snapshot of metabolic potentials in wintertime eutrophic sediments of Lake Taihu inferred from metagenome-assembled genomes
Source: Front Microbiol. 2026 Sep 28;17:1929411. doi: 10.3389/fmicb.2026.1929411 (PMC13656212; doi:10.3389/fmicb.2026.1929411)
Supplement: Supplementary file 1 [file Supplementary_file_1.DOCX]

**A Metagenomic Snapshot of Metabolic Potentials in Wintertime Eutrophic Sediments of Lake Taihu Inferred from Metagenome-Assembled Genomes**

**Qi Wei^1,2^, Yajie Li^1^, Aidong Ruan^1 *^**

^1^College of Hydrology and Water Resources, Hohai University, Nanjing, 210098, China

^2^PowerChina Guiyang Engineering Corporation Limited, 550081, China

*** **Corresponding author**: Aidong Ruan ([adruan@hhu.edu.cn](mailto:adruan@hhu.edu.cn))

**Figure S1 Functional profile of the dereplicated MAG community as assessed by METABOLIC.** Metabolic weight (MW) scores represent the functional enrichment of each pathway across the entire community. The percentage contribution of each MW score indicates the proportional functional input of individual phyla to the overall community-level metabolic potential.
